# Effect of nucleolin on adriamycin resistance via the regulation of B‐cell lymphoma 2 expression in Burkitt's lymphoma cells

**DOI:** 10.1002/jcp.28833

**Published:** 2019-05-24

**Authors:** Xuqiao Mei, Yanxin Chen, Donghui Gan, Yingyu Chen, Lingyan Wang, Yanqin Cao, Zhengjun Wu, Weijuan Liu, Chenxing Zhao, Minhui Lin, Ting Yang, Jianda Hu

**Affiliations:** ^1^ Fujian Provincial Key Laboratory of Hematology Fujian Institute of Hematology, Fujian Medical University Union Hospital Fuzhou Fujian China; ^2^ Department of Clinical Laboratory The Affiliated Zhangzhou Municipal Hospital, Fujian Medical University Zhangzhou Fujian China; ^3^ Department of Hematology The Affiliated Hospital of Putian University Putian Fujian China

**Keywords:** adriamycin resistance, Bcl‐2, Burkitt's lymphoma, CA46, nucleolin

## Abstract

Nucleolin (NCL, C23) is an important nucleocytoplasmic multifunctional protein. Due to its multifaceted profile and high expression in cancer, NCL is considered to be a marker of drug resistance associated with chemotherapy. However, the biochemical mechanisms in which NCL suppresses drug sensitivity in several cancers have yet to be fully elucidated. This study aims to explore the effect of NCL on drug sensitivity and its potential mechanism in CA46 Burkitt's lymphoma (BL) cells. CA46 BL cells were transfected with lentiviruses carrying the NCL gene (CA46‐NCL‐overexpression, CA46‐NCL‐OE), or shRNA sequences that target the endogenous NCL gene (CA46‐NCL‐knockdown, CA46‐NCL‐KD). Adriamycin (ADM) IC50 levels for CA46‐NCL‐overexpressed (OE), CA46‐NCL‐OE control (OEC), CA46‐NCL‐knockdown (KD), and CA46‐NCL‐KD control (KDC) cells were 0.68 ± 0.06 μg/ml, 0.68 ± 0.06 μg/ml, 0.68 ± 0.06 μg/ml, and 0.30 ± 0.04 μg/ml, respectively. Apoptosis rates were significantly increased following NCL KD, whereas the opposite effect was noted in OE cells. A significant reduction of B‐cell lymphoma 2 (Bcl‐2) mRNA and protein levels in KD cells was observed, while OE cells displayed the opposite effect. The stability of Bcl‐2 mRNA was influenced by NCL levels, the half‐life of which was extended after NCL‐OE, whereas it was reduced in KD cells. Finally, results of RNA‐immunoprecipitation assays indicated that NCL could bind to Bcl‐2 mRNA in CA46 cells. Taken together, these results suggested that NCL could mediate Bcl‐2 expression and stability, and thus enhance ADM resistance in CA46 BL cells.

## 1 INTRODUCTION

Burkitt's lymphoma (BL) is a high‐grade, rapidly growing and aggressive B‐cell non‐Hodgkin's lymphoma. There are three forms of BL: those endemic to Africa, sporadic forms, and those associated with immunodeficiency states (Said, Lones, & Yea, 2014). Currently, chemotherapy remains the main approach for BL treatment. However, chemotherapeutic drug resistance during treatment leads to treatment failure and inferior prognosis, and has therefore become a significant challenge (Richter‐Larrea et al., 2010).

Nucleolin (NCL, C23) is an important nucleocytoplasmic multifunctional protein that is abundantly expressed in the nucleolus, nucleoplasm, cytoplasm, and cell membrane (Srivastava & Pollard, 1999). The three main structural domains of this protein enable its interaction with different proteins and RNA sequences. NCL has been found to be involved in a variety of cellular functions, such as cell proliferation, angiogenesis, apoptosis regulation, stress response, and microRNA processing (Jia, Yao, Zhao, Guan, & Gao, 2017). Moreover, NCL is responsible for the stability of various protein molecules involved in the apoptosis process (C. Y. Chen et al., 2000; Sengupta, Bandyopadhyay, Fernandes, & Spicer, 2004). The increased expression of plasma membrane NCL has been observed in a series of tumor cells, which has also been found to contribute to oncogenesis (El Khoury et al., 2010; Hoja‐Lukowicz et al., 2009; Lv et al., 2015; Reyes‐Reyes & Akiyama, 2008). Moreover, NCL expression is significantly raised in HL‐60/ADR (HL‐60 adriamycin [ADM] resistant cells) compared to parental HL‐60 leukemia cells (Hu et al., 2011). Due to its multifaceted profile and high expression in tumor cells—particularly drug‐resistant tumor cells—NCL may act as an oncogene and drug‐resistant pusher. However, the internal biochemical mechanisms that elevate nucleolin and are involved in cellular drug resistance have yet to be elucidated.

The B‐cell lymphoma 2 (Bcl‐2) gene is highly expressed in more than half of cancer cell types and encodes a 29‐kD protein that functions as an inhibitor of cell apoptosis (Amundson et al., 2000). It has been demonstrated that Bcl‐2 was overexpressed, leading to apoptosis inhibition during the development of a variety of tumors (Buggins & Pepper, 2010; Khan & Kahl, 2018; Kulsoom et al., 2018). Studies have clarified that Bcl‐2 protein overexpression results in an apoptosis inhibition effect, which subsequently leads to chemotherapy resistance in hematological tumors and other solid tumors (Pan et al., 2015; Ruefli‐Brasse & Reed, 2017; Weller, Malipiero, Aguzzi, Reed, & Fontana, 1995).

High NCL and Bcl‐2 expression in tumor cells, along with their involvement in cell apoptosis, suggests that they play a relevant role in tumor cell drug resistance. Recent studies have found NCL to be a Bcl‐2 mRNA stabilizing protein in HL‐60 leukemia cells (Otake, Sengupta, Bandyopadhyay, Spicer, & Fernandes, 2005). This protein binds speciﬁcally to the ARE‐1 instability element in the 3′‐untranslated region (3′‐UTR) of the Bcl‐2 mRNA to protect it from ribonuclease degradation. However, the effects of the differential expression of NCL in BL cells with regard to chemotherapeutic drug resistance are unclear. We hypothesize that the regulation of Bcl‐2 mRNA stability by NCL may contribute to drug resistance in BL. To address this hypothesis, we established NCL stable expressing cell models using lentivirus‐mediated NCL overexpression (OE) or NCL knockdown (KD) in CA46 BL cells, and thus determined that Bcl‐2 (on a molecular level) likely involved in the contribution of NCL with respect to drug resistance.

## 2 MATERIAL AND METHODS

### 2.1 Cell culture

The human BL cell line CA46, with STR authentication and no mycoplasma contamination, was maintained at the Fujian Institute of Hematology (Fuzhou, China). CA46 cells were grown at 37°C under a 5% CO_2_ in a humidified atmosphere (HealthForce, Shanghai, China) in RPMI 1640 medium (Hyclone, UT) and supplemented with 10% heat‐inactivated fetal bovine serum (Hyclone).

### 2.2 Lentivirus‐mediated NCL KD and OE

Human NCL shRNA and the control scramble shRNA oligonucleotides, respectively, were cloned into pGCL‐tet‐puro‐EGFP vectors (Genechem, Shanghai, China). 5.0 × 10^5^ CA46 cells were transfected with the lentivirus at a final concentration of 300 multiplicity of infection (MOI). Posttransfected cells were named CA46‐NCL‐KD and CA46‐NCL‐KD control (KDC) cells. These were subsequently maintained at 1 μg/ml puromycin and expressed shRNA after tetracycline exposure. The NCL KD sequences were as follows:

NCL shRNA:

sense: 5′‐CCGGCGGTGAAATTGATGGAAATAACTCGAGTTATTTCCATCAATTTCACCGTTTTTg‐3′,

antisense: 5′‐AATTCAAAAACGGTGAAATTGATGGAAATAACTCGAGTTATTTCCATCAATTTCACCG‐3′.

Scramble shRNA:

sense: 5′‐CCGGTTCTCCGAACGTGTCACGTTTCAAGAGAACGTGACACGTTCGGAGAATTTTTG‐3′,

antisense: 5′‐AATTCAAAAATTCTCCGAACGTGTCACGTAAGTTCTCTACGTGACACGTTCGGAGAA‐3′.

The full‐length NCL gene was amplified and cloned into a GV113‐red fluorescent protein (RFP; Genechem) expression vector. 1.0 × 10^5^ CA46 cells were transfected with the NCL‐expressing vector and its control‐empty vector, respectively, at a 100 MOI. Posttransfected cells were named CA46‐NCL‐OE and CA46‐NCL‐OE control (CA46‐NCL‐OEC).

The primers used to amplify the NCL gene were as follows:

NCL OE:

sense: 5′‐GAGGATCCCCGGGTACCGGTCGCCACCATGGTGAAGCTCGCGAAGGCAG‐3,

antisense: 5′‐TCCTTGTAGTCCATACCTTCAAACTTCGTCTTCTTTCC‐3.

### 2.3 MTS assay

ADM was purchased from Sigma‐Aldrich (St. Louis, MO), and then diluted with a stock concentration of 2 mg/ml. The ADM sensitivity of CA46 cells was measured using the MTS assay (CellTiter 96® AQueous One Solution; Promega, Madison, WI) with several minor modifications. CA46 cells were adjusted to 2 × 10^4^ each well in 96‐well plates, and cultured in different concentrations of ADM (0–3.2 µg/ml) for 44 hrs. A total of 20 µl of MTS solution was added to each well, and the samples were incubated for 4 hrs. The plates were read on an Elx808 Absorbance Microplate Reader (BioTek, Winooski, UT) at 490 nm. The proliferation inhibitory rate (%) was calculated as: [1‐(absorbance of ADM treated group/absorbance of untreated group)] × 100. The results are presented as growth‐inhibitory curves. The 50% inhibitory concentration (IC50) was calculated using SPSS19.0. software (IBM, Armonk, NY).

### 2.4 Analysis of apoptosis using flow cytometry and 4′,6‐diamidino‐2‐phenylindole (DAPI) staining

Annexin V–fluorescein isothiocyanate (FITC), annexin V–PE, and 7‐aminoactinomycin D (7‐AAD) were purchased from BD Biosciences (San Jose, CA). NCL‐KD and KDC cells were resuspended in binding buffer and stained with annexin V–PE and 7‐AAD, according to the manufacturer's protocol (BD Biosciences), because they expressed green fluorescent protein. The CA46‐NCL‐OE and CA46‐NCL‐OEC cells included the RFP gene, and therefore these cells were stained with annexin V–FITC and 7‐AAD, also in accordance with the manufacturer's protocol (BD Biosciences). The CA46 cells were treated as control cells. The apoptotic cells (annexin V^+^/7‐AAD^–^) were detected and quantified using BD FACSCanto™IIanalysis (BD Biosciences). Flow cytometry data were analyzed using FlowJo7.6.1 (Tomy Digital Biology, Tokyo, Japan).

For DAPI staining assay, the CA46 cells and posttransduction cells were suspended in phosphate‐buffered saline (PBS) and stained with DAPI, according to the manufacturer's protocol (Beyotime, Hangzhou, China). The apoptotic cells were imaged using a fluorescence microscope.

### 2.5 Quantitative real‐time PCR (qPCR)

Total RNA was extracted using a TRIzol reagent (Life Tech Invitrogen, Carlsbad, CA) and subjected to reverse‐transcription with a GoScript™ Reverse‐Transcription System kit, according to the manufacturer's protocol (Promega). cDNA was amplified using a SYBR Green PCR master mix with a GoTaq® qPCR Master Mix kit (Promega). The PCR was performed with a PE ABI‐7500 Real‐Time PCR system (Applied Biosystems, Carlsbad, CA). The results were analyzed using the ∆CT and −2^∆∆CT^ quantification method in the ABI‐7500 software (Applied Biosystems), as previously described (Lin et al., 2013). The gene primers sequence were as follows: Bcl‐2: forward: 5′‐GAACTGGGGGAGGATTGTGG‐3′, reverse: 5′‐CCGTACAGTTCCACAAAGGC‐3′; NCL: forward: 5′‐CTTGCTGTTGTGGATGTC‐3′, reverse: 5′‐CATGGCGTCTTCAAACAC‐3′; GAPDH: forward: 5′‐CAGCTGGCCATCGAGATCA‐3′, reverse: 5′‐TCCAGTCTCTGAGCCTCATGC‐3′.

### 2.6 Determination of Bcl‐2 mRNA stability with actinomycin D (Act D)

The CA46 cells were seeded at a density of 2 × 10^5^ cells/ml, and 2 µg/ml Act D (Sigma‐Aldrich) was added. The final ethanol concentration did not exceed 0.5%. The cells were cultured at 37°C in 5% CO_2_, and subsequently harvested starting at 0 hrs, and then for every 60 min for the following 8 hrs. At this concentration, Act D did not induce DNA fragmentation during the given time period. Total RNA was isolated and the level of Bcl‐2 mRNA was detected using qPCR.

### 2.7 Western blot analysis

Western blot was performed, as previously described (Hu et al., 2011). The primary antibodies used for western blot analysis were as follows: NCL, Bcl‐2, β‐actin (Santa Cruz, CA), caspase3, and cleaved caspase3 (Cell Signaling Technology, Danvers, MA). Anti‐mouse and anti‐rabbit secondary antibodies were used in accordance with the manufacturer's recommendation (Santa Cruz). Proteins were detected using a chemiluminescent substrate (ECL; Beyotime).

### 2.8 Coimmunoprecipitation of NCL protein and Bcl‐2 mRNA complexes

The immunoprecipitation of NCL protein and Bcl‐2 mRNA complexes was undertaken according to the instructions provided by the manufacturer (EZ‐Magna RIP Kit, Millipore, Billerica, MA). Briefly, CA46 cells were lysed in RIPA lysis buffer. A total of 5 µg of anti‐NCL antibody (Santa Cruz) was cross‐linked with protein A/G magnetic beads, for which mouse IgG was used as a control antibody. Then, the cell lysates were incubated with the cross‐linked magnetic beads overnight at 4°C in a RNA‐immunoprecipitation (RIP) buffer. The magnetic beads were eventually washed off from any unbound material. Proteinase K buffer was used to digest the proteins in magnetic beads. The RNA was extracted and purified with a phenol:chloroform:isoamyl alcohol mixture. The cDNA was synthesized using random hexamer primers and reverse transcriptase, as described above. RT‐PCR was conducted under the following conditions: initial denaturation for 10 min at 95°C, followed by 40 cycles of denaturation at 95°C for 15 s and amplification at 60°C for 60 s. The PCR products were separated on 1.5% agarose gels and stained with GoldView, and the results were quantified by determining the band intensity using Bio‐Rad ChemiDoc™ Touch (Bio‐Rad Laboratories, Hercules, CA).

### 2.9 Statistical analysis

The results are presented as the mean ± *SD* of the three independent experiments. The differences among the groups were analyzed using a one‐way analysis of variance with Tukey's test for post hoc analysis. The significance was evaluated using GraphPad Prism 6.0 software (Version6.0; GraphPad, San Diego, CA). A value of 0.05 or lower was considered to represent a statistically significant difference between the groups.

### 2.10 Statement of ethics

Neither human studies nor animal studies were applied in this study.

## 3 RESULTS

### 3.1 ADM resistance was affected by NCL in CA46 cells

**Figure 1:**
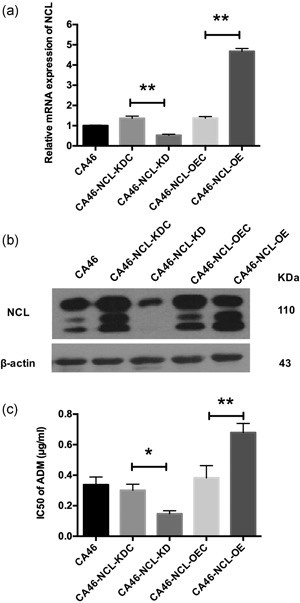
Effect of NCL KD and OE on drug sensitivity of adriamycin in human Burkitt's lymphoma cell line CA46. CA46, nontransduced CA46 cells; CA46‐NCL‐KDC, CA46 cells transduced with lentivirus‐mediated scramble shRNA as control; CA46‐NCL‐KD, CA46 cells transduced with lentivirus‐mediated NCL shRNA; CA46‐NCL‐OEC, CA46 cells transduced with empty lentiviral vector as control; CA46‐NCL‐OE, CA46 cells transduced with lentivirus‐mediated NCL overexpression. (a) qRT‐PCR was applied to detect NCL mRNA expression. Relative mRNA expression was normalized to CA46. (b) Western blot analysis was applied to detect NCL protein expression. β‐Actin was used as a loading control. (c) Drug sensitivity of adriamycin was determined using the MTS assay. The results were are presented as IC50 values for each group. Data were are shown as mean ± *SD* for at least three independent experiments. KD, knockdown; NCL, nucleolin; OEC, overexpression control; qRT‐PCR, quantitative reverse‐transcription polymerase chain reaction; shRNA, short hairpin RNA. *p < .05, **p < .01

To investigate the effect of NCL on drug resistance in BL cells, a CA46‐NCL‐OE and KD cell model were established by lentivirus transfection, respectively, and subsequently validated at both the mRNA and protein level (Figure 1a,b). The data indicated that the CA46, OEC, and KDC groups exhibited similar NCL levels, whereas in KD cells, the mRNA level and protein expression of full‐length NCL (110 kD) and its proteolysis products (Otake et al., 2007; Soundararajan, Chen, Spicer, Courtenay‐Luck, & Fernandes, 2008) were markedly reduced (*p* < .01; Figure 1a,b). In OE cells, mRNA and protein expression were significantly higher than in OEC cells (*p* < .01; Figure 1a,b). Furthermore, cell viability studies using the MTS test reveal that OE cells exhibited significantly higher IC50 for ADM compared to OEC cells (*p* < .01; Figure 1c). Specifically, the IC50 increased from 0.39 ± 0.08 μg/ml in OEC cells to 0.68 ± 0.06 μg/ml in OE cells. In contrast, KD cells exhibited significantly lower IC50 compared to both KDC cells and CA46 cells (*p* < .05; Figure 1c). The IC50 of CA46 cells was 0.34 ± 0.05 μg/ml, whereas the IC50s of the KDC and KD cells were 0.30 ± 0.04 and 0.15 ± 0.02 μg/ml, respectively (Figure 1c). Thus, the data suggested that NCL played a role in promoting cellular drug resistance.

### 3.2 NCL played a role in apoptosis induction in CA46 cells

**Figure 2:**
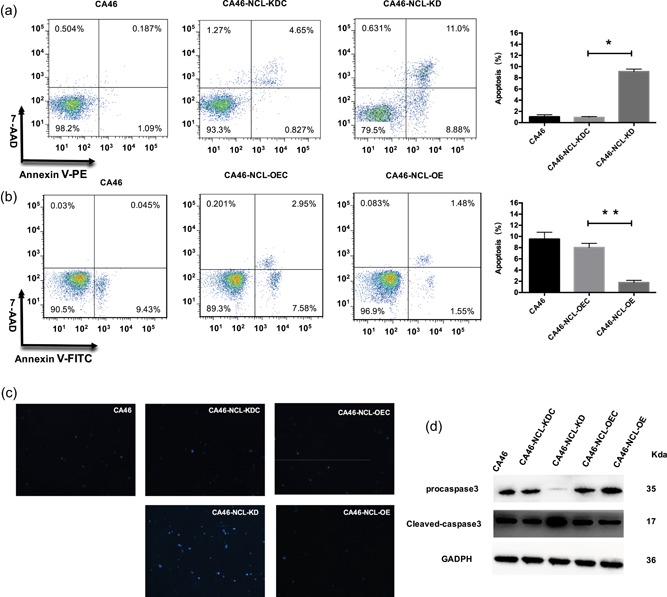
Effect of NCL knockdown and overexpression on induction of apoptosis in CA46 cells. The induction of apoptosis was determined using flow cytometry. (a) Knockdown of NCL increased the induction of apoptosis in CA46 cells. (b) Overexpression of NCL decreased the induction of apoptosis in CA46 these cells. The data indicate mean ± *SD* for at least three independent experiments. (c) Apoptosis was measured by DAPI staining. (d) Caspase3 protein were examined by western blot analysis. DAPI, 4′,6‐diamidino‐2‐phenylindole; NCL, nucleolin. *p  < .05, **p < .01 [Color figure can be viewed at wileyonlinelibrary.com]

Cell apoptosis was detected in CA46‐NCL‐OE and CA46‐NCL‐KD cells, and their corresponding control groups. Using the annexin V assay and the DNA intercalator 7‐AAD staining assay, KD cells exhibited more intense apoptosis than their control cells (*p* < .05; Figure 2a). Conversely, apoptosis rates decreased after NCL OE (*p* < .01; Figure 2b). Cell apoptosis was also confirmed by DAPI staining assay and caspase3 protein test. Nucleus DAPI staining was increased in KD cells, but decreased in OE cells. Caspase3 were activated in KD cells (Figure 2c,d). Thus, the results suggested that NCL was involved in apoptosis in CA46 cells.

### 3.3 NCL affected expression levels of Bcl‐2 mRNA and protein

**Figure 3:**
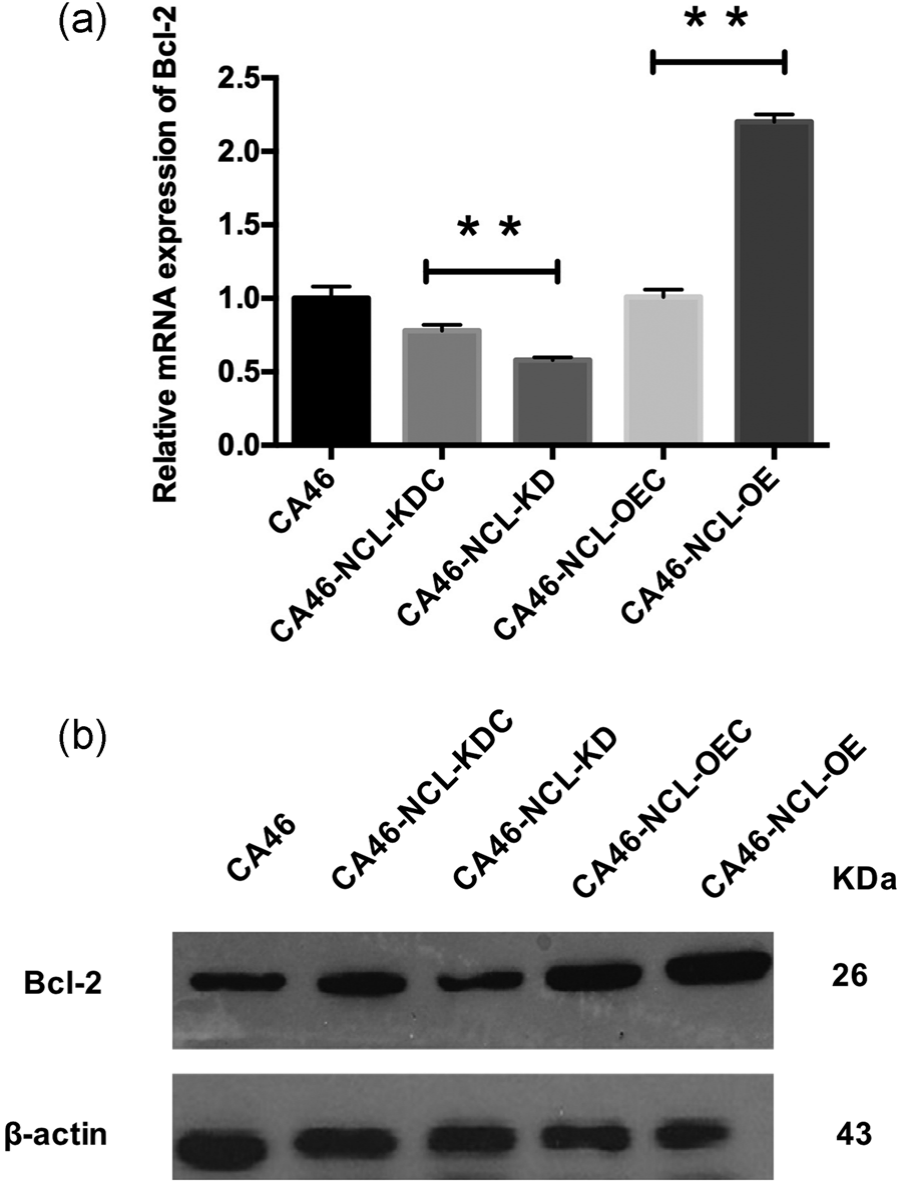
Effect of NCL knockdown and overexpression on Bcl‐2 mRNA and protein expression in CA46 cells. (a) qRT‐PCR was applied to detect Bcl‐2 mRNA. Relative mRNA expression was normalized to CA46. (b) Western blot analysis was applied to detect Bcl‐2 protein expression levels. Data indicated mean ± *SD* for at least three independent experiments. Bcl‐2, B‐cell lymphoma 2; NCL, nucleolin; qRT‐PCR, quantitative reverse‐transcription polymerase chain reaction. *p < .05, **p < .01

OE of Bcl‐2 can confer cell resistance to a variety of anticancer drugs through the inhibition of apoptosis (Pan et al., 2015). To investigate whether BCL‐2 is involved in the apoptosis inhibition effect of NCL OE on CA46 cells, the mRNA and protein levels of Bcl‐2 were detected using qPCR and western blot assays, respectively. As expected, Bcl‐2 protein levels (in accordance with mRNA levels) increased after NCL OE and were reduced following NCL KD (*p* < .01; Figure 3a,b). These results indicated that Bcl‐2 expression was likely regulated by NCL.

### 3.4 Effect of NCL in Bcl‐2 mRNA stability

**Figure 4:**
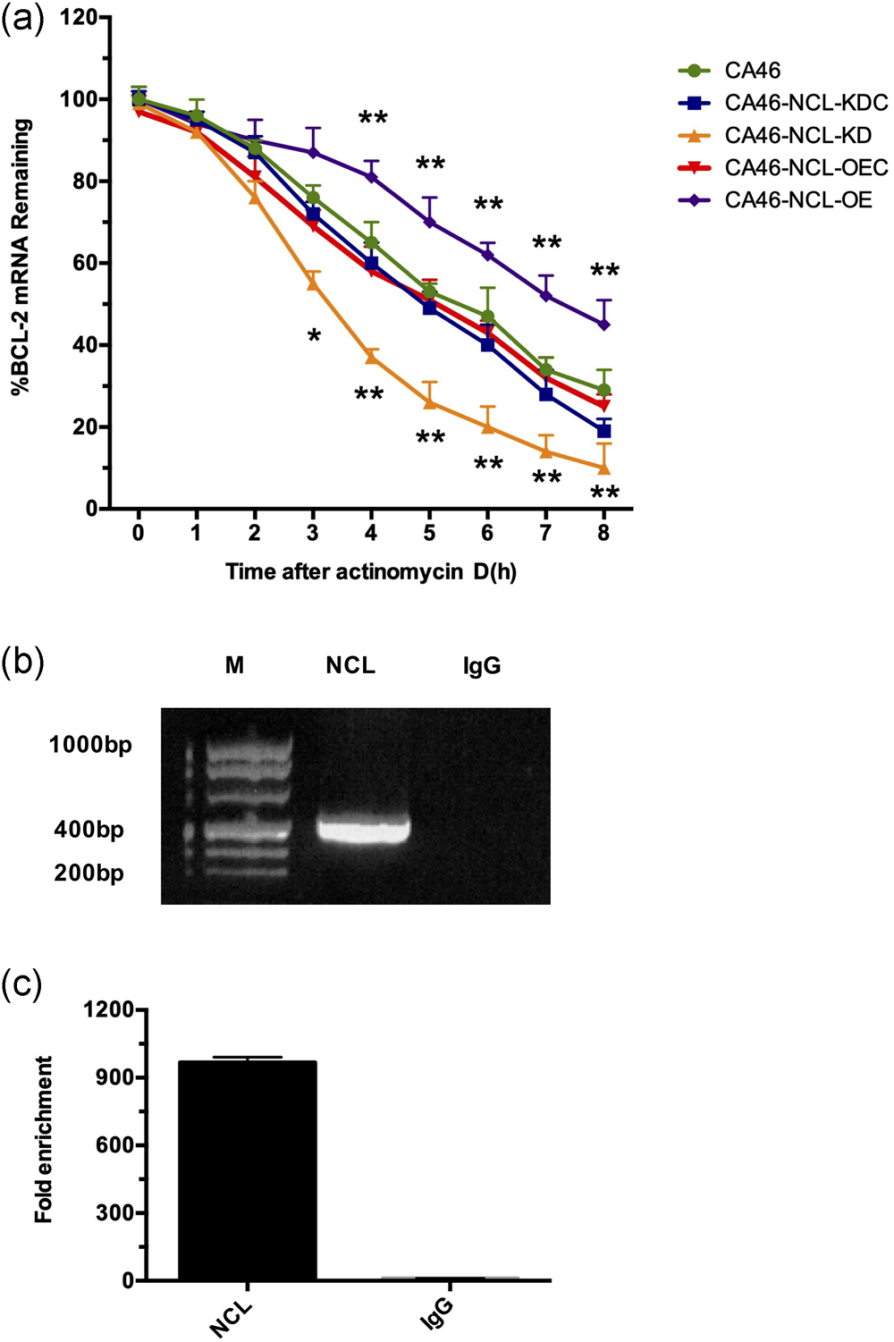
NCL modulated the stability of Bcl‐2 mRNA via binding directly to the mRNA transcript. (a) Decay of Bcl‐2 mRNA in the different CA46 groups following treatment with actinomycin D (2 µg/ml) for the indicated time periods. qRT‐PCR was performed to detect Bcl‐2 mRNA. The results were are expressed as percentage of the Bcl‐2/β‐actin ratio at the indicated time points compared with the Bcl‐2/β‐actin mRNA ratio at time 0 hr (CA46‐NCL‐KD vs. CA46‐NCL‐KDC, CA46‐NCL‐OE vs. CA46‐NCL‐OEC, *p < .05, **p < .01). (b) Bcl‐2 mRNA was enriched by RIP assay with an anti‐NCL antibody. Bcl‐2 mRNA was measured by qRT‐PCR. M: molecular marker. NCL: Bcl‐2 mRNA was immunoprecipitated with an anti‐NCL antibody. IgG: negative control; Bcl‐2 mRNA was immunoprecipitated with a normal mouse IgG antibody. Data indicated mean ± *SD* for at least three independent experiments. Bcl‐2, B‐cell lymphoma 2; KDC, knockdown control; NCL, nucleolin; OEC, overexpression control; qRT‐PCR, quantitative reverse‐transcription polymerase chain reaction; RIP, RNA‐immunoprecipitation [Color figure can be viewed at wileyonlinelibrary.com]

To uncover the biological mechanism underlying the expression regulation effect of NCL on Bcl‐2, an Act D test was used to determine whether NCL modulated Bcl‐2 expression by mRNA stability regulation. The RNA polymerase inhibitor Act D binds DNA in a guanine‐dependent manner and interferes with RNA polymerase and DNA topoisomerase enzymes. Whereas, at low concentrations (up to 5 µg/ml), it is a strong and specific inhibitor of ribosomal gene transcription (Pan et al., 2015). A significant decrease of Bcl‐2 mRNA in KD cells was noted compared to KDC cells at 3, 4, 5, 6, 7 and 8 hrs, following incubation with Act D (Figure 4a). At all‐time points (1–8 hrs), groups KDC, OEC, and CA46 exhibited a similar decline tendency in Bcl‐2 mRNA levels, which were lower and higher than the corresponding levels in the OE and KD cells, respectively (Figure 4a). The half‐life of Bcl‐2 mRNA was extended from 5.22 ± 0.21 hrs in OEC cells to 6.95 ± 0.13 hrs in OE cells, whereas it declined from 4.86 ± 0.15 hrs in KDC cells to 3.23 ± 0.18 hrs in KD cells (Figure 4a). The results indicated that Bcl‐2 mRNA stability increased with NCL OE, and decreased with NCL KD. Thus, NCL was confirmed to positively impact Bcl‐2 mRNA stability.

### 3.5 Bcl‐2 mRNA bond with NCL protein

It was verified that NCL can induce the stabilization of Bcl‐2 mRNA by binding with Bcl‐2 ARE‐1 mRNA. Further experiments were performed to clarify whether the NCL protein could bind with Bcl‐2 mRNA, and thus enhance Bcl‐2 mRNA stability in BL cells. Results of RIP assays revealed a significant enrichment of Bcl‐2 mRNA in NCL immunoprecipitated complexes compared with anti‐IgG control (Figure 4b). Thus, the results revealed that the NCL protein physically bond with Bcl‐2 mRNA in BL cells.

## 4 DISCUSSION

Treatment failure of BL is often caused by resistance to chemotherapeutic drugs (Richter‐Larrea et al., 2010). Mechanisms of drug resistance that have recently been clarified are as follows: gene aberrations, apoptosis regulation abnormal, and bone marrow microenvironment change (Dlugosz & Janecka, 2016; Morabito et al., 1997; Tsuchiya et al., 2015). In this study, we found that NCL OE can promote drug resistance in BL, a result which may be related to the stabilization of Bcl‐2 mRNA and decreased induction of apoptosis.

NCL is closely related to the proliferation and survival of cancer cells. Several studies have suggested that this protein exhibits a high expression in malignant cells, most notably in tumor cells (Pichiorri et al., 2013; Shen et al., 2014; Zhao et al., 2013). Clinically, overexpressed NCL in relapse/refractory acute leukemia patient's cells with poor prognosis was found in our group's study (Hu et al., 2011). It has been reported that high NCL expression level in AML blasts is associated with poor overall survival rates (Marcel et al., 2017). Biologically, NCL is known to act in proliferation promotion and drug resistance evolution in acute leukemia cells (Westmark & Malter, 2001). It has been suggested that chemotherapeutic drug‐induced apoptosis in HL‐60 cells is preceded by the Bcl‐2 mRNA destabilization and the downregulation of Bcl‐2 mRNA and protein levels (Bandyopadhyay, Sengupta, Fernandes, & Spicer, 2003; Riordan, Foroni, Hoffbrand, Mehta, & Wickremasinghe, 1998). In addition, Westmark and Malter have suggested that NCL downregulation is an essential step for the antitumor effects of chemotherapeutic drugs (Westmark & Malter, 2001). Our previous studies had noted that NCL is overexpressed in the ADM resistance CA46 BL cells (unpublished data). The current study is a part of our group's research, which worked on clarifying the mechanisms of drug resistance in hematological malignancies. The relationship between NCL and ADM resistance was evidenced from these results. The present results demonstrate the involvement of NCL in the drug resistance of BL cells, due to the fact that NCL OE reduced ADM sensitivity in CA46 cells, whereas the opposite effect was noted in NCL KD cells.

The association between NCL and apoptosis induction has been extensively investigated in a range of cancerous and noncancerous human cell lines. The downregulation of NCL can be accomplished using ADP‐sensitized human umbilical vein endothelial cells (HUVECs) to cisplatin‐induced cell death via the downregulation of Bcl‐2 expression (Wang et al., 2014). Moreover, the NCL inhibitor AS1411 inhibited the proliferation of human glioma and breast cancer cells via the destabilization and downregulation of Bcl‐2 mRNA (Y. Cheng et al., 2016; Soundararajan et al., 2008). In CLL cells, loss of NCL activates Fas‐mediated apoptosis. In B‐cell lymphoma, NCL KD sensitized cells to FasL‐induced apoptosis and induced phosphorylation of H2AX and B‐cell lymphoma survival (Jain et al., 2017; Kaufman et al., 2015; Wise et al., 2013). One study notably investigated perinucleolar localization and the binding of NCL to the CCND1 allele, which encoded the transcription of the cyclin D1 protein in mantle cell lymphoma (Allinne et al., 2014). Nevertheless, with regard to BL and NCL, current studies are limited. NCL was first identified as an apoptosis‐associated protein in human BL cells in 1998 (Brockstedt et al., 1998). The present study provides a valuable mechanistic approach to explain the influence of NCL in BL cellular viability and sensitivity to ADM treatment. OE of NCL in BL cells decreased apoptosis and drug sensitivity, whereas KD of NCL resulted in the opposite effect. Expression level of Bcl‐2 mRNA and protein displayed a positive correlation with NCL level occurring via the modulation of the Bcl‐2 protein and mRNA expression.

In addition, the present analysis reveals the functional interaction of NCL proteins with Bcl‐2 mRNA in CA46 cells. For this purpose, Act D was initially used to bind to DNA, thus preventing the synthesis of RNA and the incorporation of nucleotide triphosphates into DNA. At this low concentration ( < 5 µg/ml), Act D did not induce DNA fragmentation during the treatment period. The comparison of the half‐life of the Bcl‐2 mRNA transcripts in CA46‐NCL‐OE and CA46‐NCL‐KD cells suggested that NCL promoted Bcl‐2 mRNA stability, and thus regulated the expression of the Bcl‐2 gene. This finding was in accordance with previous studies that have examined in different cell types (Kito et al., 2003; Otake et al., 2005; Sengupta et al., 2004). Using the Act D test, our study confirmed that NCL modulated Bcl‐2 expression via mRNA stability regulation. Moreover, we demonstrated a physical binding of NCL protein and Bcl‐2 mRNA in BL cells. Previous studies have found that the NCL protein speciﬁcally bonds to the ARE‐1 instability element in the 3′‐UTR of the Bcl‐2 mRNA to protect it from ribonuclease degradation (Otake et al., 2005). Furthermore, immunoprecipitation assays have indicated that the binding of NCL and Bcl‐2 mRNA transcripts enhances Bcl‐2 stability, most likely by protecting the mRNA from exosome recruitment and subsequent decay (Ishimaru et al., 2010). While previous studies have determined the binding site of NCL and Bcl‐2 mRNA, our study clarified the modulation of NCL on the stability of BCL mRNA, the clue of which NCL modulates the stability of Bcl‐2 mRNA by directly binding to the mRNA transcript. The above results revealed the location of the binding site and the effect of NCL on Bcl‐2.

In conclusion, our study has demonstrated that high NCL expression promotes drug resistance in BL, which may be related to the stabilization of Bcl‐2 mRNA and the decreased induction of apoptosis. Importantly, NCL can bind directly to Bcl‐2 mRNA, thus modulating its stability. The data provide insight into the regulation of cancer aggressiveness and drug sensitivity via the inhibition of NCL expression in tumors. Thus, NCL could be used as a potential therapeutic target in BL.

## ACKNOWLEDGMENTS

This study was supported by National Natural Science Foundation of China (81470326), Construction Project of Fujian Medical Center of Hematology (Min201704), Special Fund from Fujian Provincial Department of Finance (2016B041), the Cooperation Project of University and Industry in Fujian Province (2017Y4005), Joint Research Project of Health and Education (WKJ2016‐2‐06), Joint Funds for the Innovation of Science and Technology, Fujian province (2016Y9029, 2016Y9032), and National Key R&D Program of China (2016YFC0902800).

## AUTHOR CONTRIBUTIONS

J. D. H., X. Q. M., and Y. X. C. conceived and designed the experiment; X. Q. M., D. H. G., Y. Y. C., and L. Y. W. operated the experiment; Y. Q. C., Z. J. W., W. J. L., and C. X. Z. analyzed the data; X. Q. M., Y. X. C., and J. D. H. wrote the paper. T. Y., M. H. L., and J. D. H. reviewed the paper. Manuscript is approved by all authors for publication.

## DATA AVAILABILITY STATEMENT

Data sharing is not applicable to this article as no new data were created or analyzed in this study.
